# Evidence of reactive oxygen species-mediated damage to mitochondrial DNA in children with typical autism

**DOI:** 10.1186/2040-2392-4-2

**Published:** 2013-01-25

**Authors:** Eleonora Napoli, Sarah Wong, Cecilia Giulivi

**Affiliations:** 1 Department of Molecular Biosciences, University of California, One Shields Ave, 1120 Haring Hall, Davis, CA, 95616, USA; 2 Medical Investigations of Neurodevelopmental Disorders (M.I.N.D.) Institute, University of California, Davis, CA, 95616, USA

**Keywords:** Autism, Mitochondria, Mitochondrial DNA, Oxidative damage, Bioenergetics

## Abstract

**Background:**

The mitochondrial genome (mtDNA) is particularly susceptible to damage mediated by reactive oxygen species (ROS). Although elevated ROS production and elevated biomarkers of oxidative stress have been found in tissues from children with autism spectrum disorders, evidence for damage to mtDNA is lacking.

**Findings:**

mtDNA deletions were evaluated in peripheral blood monocytic cells (PBMC) isolated from 2–5 year old children with full autism (AU; n = 67), and typically developing children (TD; n = 46) and their parents enrolled in the CHildhood Autism Risk from Genes and Environment study (CHARGE) at University of California Davis. Sequence variants were evaluated in mtDNA segments from AU and TD children (n = 10; each) and their mothers representing 31.2% coverage of the entire human mitochondrial genome. Increased mtDNA damage in AU children was evidenced by (i) higher frequency of mtDNA deletions (2-fold), (ii) higher number of GC→AT transitions (2.4-fold), being GC preferred sites for oxidative damage, and (iii) higher frequency of G,C,T→A transitions (1.6-fold) suggesting a higher incidence of polymerase gamma incorporating mainly A at bypassed apurinic/apyrimidinic sites, probably originated from oxidative stress. The last two outcomes were identical to their mothers suggesting the inheritance of a template consistent with increased oxidative damage, whereas the frequency of mtDNA deletions in AU children was similar to that of their fathers.

**Conclusions:**

These results suggest that a combination of genetic and epigenetic factors, taking place during perinatal periods, results in a mtDNA template in children with autism similar to that expected for older individuals.

## Background

The human mitochondrial genome is a 16.5-kb circular, double stranded DNA that encodes thirteen polypeptides of the mitochondrial respiratory chain, twenty-two transfer RNAs and two ribosomal RNAs required for protein synthesis. The mitochondrial DNA (mtDNA) consists of a heavy (H) and a light (L) strand, in accord with its G and T base composition. mtDNA is particularly susceptible to mutations because of the high level of reactive oxygen species (ROS) (including superoxide anion, hydrogen peroxide, hydroxyl radical and peroxynitrite) generation in this organelle [1,2], and lack of introns or histones, coupled with a low level of DNA repair [3].

Damage to mtDNA, elicited by ROS continually generated in mitochondria, may result from defective replication and/or repair of mtDNA of primary (genetic) [4] or secondary (for example, oxidative damage to single base pairs inflicted by ROS) [5,6] origins. ROS-mediated damage is characterized by a variety of lesions to DNA in general [7,8] and to mtDNA in particular [9], including single- and double-stranded DNA breaks, abasic sites, and oxidized bases (reviewed in [10-13]). Considering that mtDNA replication occurs as a coupled leading and lagging strand replication pathway, the H strand DNA exists for extended periods in the single-stranded form in this asymmetric mode of mtDNA replication, favoring damage to the exposed bases not protected by the complementary DNA strand [14]. In this regard, mtDNA damage is asymmetric resulting in the majority of single and multiple mtDNA deletions occurring between the *O*_H_ and *O*_L_[15]. In addition, cytosine deamination to uracil would be expected to be an asymmetric process because it occurs > 100-fold more rapidly in single-stranded DNA, as it would be when mtDNA is temporarily exposed during ongoing replication and transcription [14]. Increased oxidative stress has been claimed as one of the features present in autism [15-19], although its precise role in the etiology of autism is still undefined. In support of this hypothesis, higher rates of mitochondrial hydrogen peroxide production (accompanied by lower activities in the pyruvate dehydrogenase complex, Complex I alone, or in combination with other Complexes) from lymphocytic mitochondria [15] and increased markers of oxidative stress (for example, increased oxidized glutathione) [19-22] had been reported in samples from individuals with autism.

There are limited studies characterizing mtDNA from children with full autism, and none evaluating the putative ROS-mediated damage to mtDNA in the index child. We evaluated the occurrence of ROS-mediated damage to mtDNA in children who met the criteria for presenting full-syndrome autism and age-matched and genetically unrelated typically developing children (TD) without a clinical diagnosis of autism or developmental delays, recruited by the CHildhood Autism Risk from Genes and Environment (CHARGE) Study at the University of California (UC) Davis [23]. To minimize invasive procedures, we evaluated the quality of mtDNA (deletions and sequence variants) in peripheral blood monocytic cells (PBMC) from children with typical autism to ascertain if (i) the diagnosis of autism segregated with a pattern consistent of increased oxidative damage to mtDNA and (ii) if the putative mtDNA damage was *de novo* (when compared to their parents living in the same household) or inherited (if outcomes followed paternal and/or maternal patterns), when compared to parents of TD or AU children. To our knowledge, there are no systematic and comprehensive studies aimed at investigating the role of ROS-mediated damage to mtDNA in children with autism.

## Methods

### Clinical selection of individuals and diagnosis

The CHARGE study is an epidemiologic case control investigation launched by the UC Davis Center for Children’s Environmental Health that has been enrolling families through the Medical Investigations of Neurodevelopmental Disorders (M.I.N.D.) Institute since 2003. The focus of the CHARGE study is on modifiable factors in autism etiology and markers of biological dysregulation that may provide mechanistic clues.

Families had been recruited from three groups: children diagnosed with autism, children diagnosed with developmental delay but not an autism spectrum disorder (ASD) or autism, and children from the general population. These children are 24 to 60 months old, and reside with a biological parent in a well-defined catchment area of over 22 counties in northern California and parts of Los Angeles County. Cases are recruited through the California Department of Developmental Services system, M.I.N.D. Institute clinics, clinician referrals, and self-referrals. General population controls are sampled from birth files with frequency-matching to the projected distribution of sex, age, and geographic area among cases of autism.

Environmental, lifestyle, reproductive, maternal medical, and detailed demographic information is collected through an extensive telephone interview with the primary caregiver. Participants classify themselves into race and ethnicity categories identical to those used in the US Census. Diagnoses are confirmed through clinical examinations using the Autism Diagnostic Inventory-Revised (ADI-R) [24], and the Autism Diagnostic Observation Schedule (ADOS) [25]. The ADI-R provides a standardized, semi-structured interview and a diagnostic algorithm for the Diagnostic and Statistical Manual of Mental Disorders-Fourth Edition (Text Revision) (DSM-IV-TR) and the International Statistical Classification of Diseases and Related Health Problems 10th Revision (ICD-10) definitions of autism [26]. The ADOS is a semi-structured, standardized assessment in which the researcher observes the social interaction, communication, play, and imaginative use of materials for children suspected of having autism and ASD. The final CHARGE study diagnosis is defined as meeting criteria on the communication, social, and repetitive behavior domains of the ADI-R and scoring at or above the cutoff for autistic disorder on the ADOS (module 1, 2 or 3). The Social Communication Questionnaire is used to screen for ASD among those recruited as developmentally delayed, or as general population controls. Children who score above the screening cutoff are fully assessed using the ADI-R and ADOS. The developmental and adaptive functions of all children are evaluated with the Mullen Scales of Early Learning [27] and the Vineland Adaptive Behavior Scales [28], and children scoring 71 or above on both these scales who do not have an autism spectrum disorder qualify as TD. The child’s medical history is taken and a developmental-behavioral pediatrician conducts an examination for physical or neurological abnormalities. Further details on the CHARGE study protocols are published elsewhere [23].

For the studies on mitochondrial DNA, we sampled 67 children with full-syndrome autism and 46 classified as TD; all of these children were genetically unrelated, both within and between diagnostic groups. We also attempted to achieve comparable age, sex, and race/ethnicity in the TD and AU groups. Demographic and clinical data from these groups are presented in Additional file 1: Tables S1 and S2. A computer-generated random sampling of 10 TD and 10 AU children was used for mtDNA sequencing. The study protocol follows the ethical guidelines of the Declaration of Helsinki [29] and was approved by the Institutional Review Board of the UC Davis School of Medicine. All subjects enrolled in the study had written informed consent provided by their parents and self-assented to participate if developmentally able.

### DNA purification, PCR amplification and sequencing

Blood samples (approximately 8 ml) were collected in a BD vacutainer CPT tube (BD Biosciences, catalog number 362753; San Jose, CA, USA). CPT tubes were kept on ice until ready to use. The CPT tubes were centrifuged at 1650 *g* for 15 minutes at 22°C. The plasma layer was removed and stored at −80°C. The lymphocytes were transferred to another tube, washed according to the manufacturer’s specifications and resuspended in 600 μl of buffer A (in mM, 220 sucrose, 50 KCl, 10 KH_2_PO_4_, 5 MgCl_2_, 1 EGTA and 10 HEPES, pH 7.5). Genomic DNA was extracted from isolated lymphocytes using Puregene kit (catalog number 158388) from Qiagen (Valencia, CA, USA). Each DNA concentration was determined by triplicate measurements of the absorbance at 260 nm using a Tecan Quantiplate plate reader (Grödig, Austria). DNA was diluted to 0.63 ng/μl and served as stock DNA template for both qPCR and sequencing. To determine sequence variants in mtDNA, nt 2995 to 5570, 7960 to 9867 and 14732 to 15419 (5,172 bp out of 16,569 bp, for a 31.2% coverage of the entire human mitochondrial genome [Genbank: NC_012920] including the following protein-encoding genes: *ATP6/8*, *ND1* and *ND2*, and parts of *CYTB*, *COX2* and *COX3*) were PCR-amplified from 10 samples randomly selected from children with AU and 10 TD children in 1 to 3-kb overlapping fragments, and the PCR products were completely sequenced. The use of large PCR products excluded the possibility that nuclear pseudogenes could complicate this analysis [30]. The sequences obtained were first compared to those recorded in extensive mitochondrial databanks [31,32]. To distinguish somatic mutations from rare germline variants, we determined the variations present in samples from randomly selected age-matched TD children. It is important to notice that the sequencing was performed to look for evidence of ROS-mediated damage and not to identify or claim any pathogenic mutation associated with autism. Sequencing primers were obtained from reference [33]. Four sets of sequencing primers were used:

13f - E, 7960–7979 ATTATTCCTAGAACCAGGCG

13r - E, 8641–8621 TGATGAGATATTTGGAGGTGG

14f - E, 8563–8581 ACAATCCTAGGCCTACCCG

14r - E, 9231–9212 GATAGGCATGTGATTGGTGG

15f - E, 9181–9198 AGCCTCTACCTGCACGAC

15r - E, 9867–9848 GGATGAAGCAGATAGTGAGG

24f - H, 14732–14752 ACTACAAGAACACCAATGACC

24r - H, 15419–15400 TGTAGTAAGGGTGGAAGGTG.

For sequencing, PCR amplification was done using the Qiagen Taq DNA polymerase (catalog number Q201203; Valencia, CA, USA) consisting of 2.5 μl of 10x Buffer (Qiagen catalog number Q201203; Valencia, CA, USA), 1.7 μl of 25 mM MgCl_2_ (Qiagen catalog number Q201203; Valencia, CA, USA), 0.5 μl of 10 mM dNTP mix (Invitrogen catalog number 10297–018; Grand Island, NY, USA) , 0.5 μl of each of the two primers, 10 μl of sample with 6 ng total of DNA, 0.2 μl of Taq enzyme and 8.1 μl of MilliQ water for the total reaction volume of 24 μl. The following cycling conditions were usedP 94°C for 3 minutes, 10 cycles of 94°C for 15 s, 65°C for 30 s (with 1°C decrease at each cycle), and 72°C for 40 s, following with 30 cycles of 94°C for 15 s, 55°C for 30 s, and 72°C for 40 s. Final elongation was done for 5 minutes at 72°C with a 15°C forever-hold step. PCR products were run on 1.3% agarose gel, excised and purified using Qiaquick Gel Extraction Kit (Qiagen, catalog number 28704, Valencia, CA, USA) according to manufacturer’s instructions, and submitted for sequencing to the UC Sequencing Core Facility on the UC Davis campus. Final readouts were analyzed using Applied Biosystems Sequence Analyzer software, and alignments of sequences were done through Invitrogen’s Vector NTI software.

### Evaluation of mtDNA deletions

The majority of mtDNA deletions involve the major arc of the mitochondrial genome between the origin of the heavy strand replication (nucleotides 110 to 441) and the origin of the light strand replication (nucleotides 5721 to 5798) [31]. In the majority of patients with single and multiple mtDNA deletions, the *ND4* (mitochondrial gene encoding for the ND4 subunit of Complex I) and/or *CYTB* (mitochondrial gene encoding for cytochrome *b*) genes present deletions whereas the *ND1* (mitochondrial gene encoding subunit ND1 in Complex I) is rarely deleted; therefore, we evaluated the ratios of *ND4/ND1* and *CYTB/ND1* gene copy number with dual-labeled probes to detect mtDNA microdeletions [34] in mtDNA from PBMC from TD children (n = 46) and children with autism (n = 67). Changes in mtDNA copy number were evaluated by dual-labeled probes using quantitative (q) PCR. The gene copy number of cytochrome *b,* ND1 and ND4 were normalized by a single-copy nuclear gene (pyruvate kinase) as explained in detail before [15]. mtDNA deletions were considered if the Z-scores were < −2SD, where the means and SD were obtained with TD values for each age and sex group.

### Statistical analyses

Experiments were run in triplicate and repeated three times in independent experiments. The percentage of individuals with mtDNA deletions was calculated using the Z-scores. The Z-scores were calculated as (x_i_-mean)/SD for each group, in which the mean and SD were obtained from TD children (for comparison of children), from TD mothers (for comparison of mothers) and TD fathers (for comparison of fathers). The cutoff for considering an outcome as either high or low was > 2SD or <−2SD respectively. The chi-square test was utilized to evaluate significance in the distribution of frequencies between groups.

## Findings

Deletions in mtDNA of TD and AU children were evaluated by qPCR using the mitochondrial gene ratios of *CYTB/ND1* and *ND4/ND1*. The percentage of TD children (n = 46) with deletions (deletion = Z-score < −2SD) encoding for *CYTB* and *ND4* was 8.7% and 6.5%, respectively (Table 1). In samples from AU children (n = 67), these outcomes were significantly higher by 2.4- and 2.3-fold, respectively (Table 1). In both groups, TD and AU children, the frequency of deletions at genes located closer to *O*_H_ (*CYTB*) relative to those located closer to the *O*_L_ (*ND4*) was 1.3- and 1.4-fold, respectively, with no difference between the groups. The higher incidence of individuals with *CYTB* deletions vs. *ND4* ones was also observed in all parents, regardless of sex or diagnosis of child (Table 1, last row). This strand asymmetry of mtDNA deletions was suggestive of ROS-mediated damage to the single-stranded state of the H strand during the asynchronous mtDNA replication.

**Table 1:** Percentage of TD and AU children and their parents with mtDNA deletions

**Mitochondrial gene**	**Individuals with mtDNA deletions**									
	**Children**			**Mothers**			**Fathers**			
	**TD**	**AU**	***P*-value**	**TD**	**AU**	***P*-value**	**TD**	**AU**	***P*-value**	
Number	46	67		37	49		37	49	
*CYTB* deletions, %	8.7	20.9	< 0.001	10.8	6.1	0.02	10.8	14.6*	0.030
*ND4* deletions, %	6.5	14.9	< 0.001	8.1	4.1	0.02	5.4	10.2**	0.009
*CYTB/ND4* fold change, %	1.3	1.4		1.3	1.5		2.0	1.4	

Individuals with a Z-score < −2SD were considered as having deletions. The mean and SD utilized for the Z-scores were obtained from TD values from each of the groups (child, mother, father). The significance shown in the Table is for each AU vs. TD comparison. *P = 0.017 and **P = 0.030 vs. mothers of AU children. *mtDNA* mitochondrial DNA; *TD* typically developing; *AU* full autism.

The extent of the mtDNA deletions at *CYTB* in AU children was 14 ± 1%, 1.5-fold greater than the corresponding TD values (9 ± 1%, *P* < 0.005) and was similar to that of older individuals, regardless of sex or the diagnosis of the child (16 ± 2%, *P* < 0.01).

To discern between *de novo* (acquired) vs. inherited deletions (from either maternal mtDNA or parental gDNA-inherited mechanisms that favor accumulation of deletions in the mtDNA), deletions in both segments of the mtDNA were evaluated in the parents of TD and AU children. The percentage of fathers of AU children with mtDNA deletions at the segments encoding for *CYTB* and *ND4* was higher than for those of TD children (1.4-fold and 1.9-fold respectively), following the pattern of AU children when compared to TD children for both genes. In contrast, mothers of AU children presented a low incidence of deletions at both segments when compared to mothers of TD children (50%) (Table 1) suggesting lower mtDNA replication.

The ratio of parents of TD children to TD children with mtDNA deletions was 1.2-fold in the segment encoding for *CYTB* (Table 1). This increase suggested an age-dependent accumulation of mtDNA deletions. Age-dependent mtDNA deletions are thought to accumulate at lower levels in human mitotic tissues than post mitotic ones because they could be lost by negative selection in rapidly dividing cells, by a higher tendency of the cells to undergo apoptosis, mitochondria being able to replicate independently of the cell cycle (avoiding being diluted out by cell division) and/or by preferential replication of deleted mtDNA [35].

To determine sequence variants that were ascribed to ROS-mediated damage in mtDNA from AU and TD children, 31.2% of the entire human mtDNA was sequenced encompassing the following protein-encoding genes: *ATP6/8*, *ND1* and *ND2*, and parts of *CYTB, COX2* and *COX3*. A relatively higher frequency of transition mutations has been reported in aerobic organisms (from 60% to 68%) [36,37] consistent with the transitions observed in this study for both children regardless of diagnosis (Table 2). Transitions (90% of base variants) affecting GC and AT pairs in TD children were 44.4% and 55.6% (Table 2). This pattern of base substitutions (90.0% transitions with 55.6% of them converting an AT to a GC pair) resembles that of the human mtDNA sequence polymorphisms reported in the MITOMAP database, which shows an enormous preponderance of transitions (88%) over transversions, with a nucleotide bias approximating that of the genome (that is, the genome is 56% A + T, and 58% of the transitions A-T→G-C). In AU children, transitions affecting GC and AT pairs were 64.7% and 35.3% indicating that CG pairs were replaced 2.5 times more often than AT pairs, which was an opposite trend to TD children (Table 2). The percentage of (G,C,T)→A transitions to the total number of transitions in AU children was 1.6 times that in TD children (Table 2). 

**Table 2:** Summary of outcomes evaluated in mtDNA from TD and AU children

**Outcomes**	**AU**	**TD**	***P*-value**
Sequence variants, number	17	31	
Transitions, %	100	90	
Transversions, %	0	10	
Type of transitions			
GC transitions, %	64.7*	44.4*	0.005
AT transitions, %	35.3*	55.6*	
G,C,T → A, % of all other transitions	89*	56*	0.001

*See text for discussion of these results.

To ascertain if the sequence variants obtained with mtDNA from AU children were *de novo* or the result of maternal inheritance, the same segments were analyzed in their mothers. Consistent with the maternal inheritance of mtDNA, all variants found in children were also observed in their mothers, regardless of diagnosis.

## Discussion

In this study, and following the model of strand asymmetry replication of mtDNA, evidence for mtDNA damage has been observed in all individuals, regardless of age and/or diagnosis. The higher frequency of deletions at the segment encoding for *CYTB* compared to *ND4* (Table 1, last row) attests for an asymmetric damage of the mtDNA when the H-strand is exposed in the single-stranded state during replication.

If the hypothesis that higher oxidative damage to mtDNA was occurring in a more exacerbated form in PBMC from AU than from TD children, then the following results would be expected: (i) higher percentage of GC transitions over AT ones because when the well-known marker of oxidative stress, 8-oxo-7,8-dihydro-2′-deoxyguanosine (8oxodG)[1,38,39], is effectively repaired and/or removed from the template [40], inducing predominantly G→T transitions by mispairing with A during DNA replication [41-43]; (ii) higher frequency of mtDNA deletions, especially at the segment encoding for *CYTB* compared to *ND4*; and (iii) higher frequency of G,C,T→A transitions because when Polγ bypasses apurinic/apyrimidinic (AP) sites, formed as a result of spontaneous deamination or oxidative lesions, incorporating mainly adenine at these positions [44].

In favor of the model of higher oxidative stress in autism, a higher frequency of GC transitions over AT ones (2 vs. 0.8) (Table 2), higher frequency of deletions (by 2-fold) (Table 1), and higher number of G,C,T→A transitions (1.3-fold those in TD children) (Table 2) were observed in AU children. The lack of G→T transitions could be explained, considering that these types of transitions are rarely observed in both *in vivo* and *in vitro* somatic sets of mtDNA point mutations [45,46]. The extent of the deletions in AU children was 1.6-fold of that in TD children, and was similar to that of all parents, suggesting more damage to their mtDNA. However, given that the percentages observed in AU children were similar to those observed in older individuals in general, and that these percentages are usually much lower than are seen in patients with mitochondrial disorders, in which deletion ≥ 60% is required to demonstrate a mitochondrial defect [47,48], it is suggested that the extent of the deletions do not seem to be pathogenic per se.

The sequence variants observed in all children were explained by the maternal inheritance of mtDNA, regardless of diagnosis (Table 2). However, given that these outcomes were different between TD child-mother vs. AU child-mother, it suggests that mothers of AU children share a DNA template consistent with a model of higher oxidative stress-mediated damage. It is interesting to note that mothers of AU children, although having more damaged mtDNA, also presented the lowest incidence of deletions when compared to age-matched groups. This might indicate a compensatory mechanism, by which a lower replicative rate might prevent additional accumulation of deletions.

Considering that a higher percentage of AU children exhibited mtDNA deletions compared to TD children, and that this pattern was also present in fathers of AU children, it is likely that a genetic predisposition to accumulate mtDNA deletions was transmitted paternally. It should be noted that paternal mtDNA deletions are not inherited but, accumulation of deletions (or the predisposition to accumulate deletions) resulting from increased ROS production, defective antioxidant/repair system, or defective clearance of damaged mitochondria, could be transmitted from either parent. Alternatively, exposure to epigenetic factors different from those to which families of TD children are exposed, or identical to those of TD families but perceived with a different genetic susceptibility [49], may have resulted in the increased mtDNA deletions observed in AU children and their fathers.

This study has several limitations that need to be considered for a proper interpretation of the results and consequences for the field of autism. First, the number of individuals on which mtDNA sequencing was performed was relatively small, although significant differences were observed between TD child-mother and AU child-mother. Second, the comparisons made in this study reached a significance at the α = 0.001 level minimizing type I errors, whereas type II errors were reduced by increasing the number of observations per group, limited only by the availability of samples. Third, children in this study had not been previously diagnosed with a genetic syndrome, nor had any indications of genetic syndromes been identified by M.I.N.D. developmental pediatricians. Nevertheless, defects (other than deletions) in genes other than those tested could have been present in these samples as recently reported in other studies of ASD [50]. Fourth, although the outcomes reported here for PBMC may represent those present in other cells more relevant to autism (for example, neurons), it is important to consider that the neuroimmune response is characterized by cross-talk between peripheral immune cells and the central nervous system, and that disruption of this process during early life may condition inflammatory responses as well as behavioral changes that persist during adulthood [51-54]. Finally, inferences about a cause-effect association between ROS-mediated mtDNA damage and typical autism are intricate because this is cross-sectional, and not a longitudinal study. In addition, several factors influence expression of mtDNA damage, for example, nuclear genetic backgrounds [55], mtDNA heteroplasmy in tissues [56], energy thresholds for a given tissue/organ [57], and epigenetic factors [58], in both affected and general healthy populations. Multiple mtDNA deletions for example, may accumulate with age in post-mitotic tissues of apparently healthy individuals [12,48,59-61], or in patients with other disorders not necessarily linked to autism, such as, inherited mutations in nuclear genes [62,63], neurodegenerative disorders [62,64], cancer [65], and diabetes [66]. Nevertheless, our study showed that mtDNA in children with autism is more damaged than in age-, sex-, and race-matched TD children, and is more similar to that of older individuals, with a mtDNA template (maternally inherited) consistent with ROS-mediated damage (based on sequence variants), and presenting a predisposition to accumulate damage (deletions) similar to that of their fathers.

## Conclusions

Several scenarios may result in increased mtDNA damage, among them: (i) higher oxidative stress not accompanied by antioxidant defenses and/or repair/maintenance of mtDNA; (ii) lower ability to clear mitochondria with damaged mtDNA; (iii) replicative advantage of deleted mtDNA over wild-type mtDNA, or (iv) a combination of any of the aforementioned possibilities.

The fact that mothers of AU children have a template with an array consistent with increased mtDNA damage, and that fathers of AU children accumulate more deletions than fathers of TD children, seems to point to a combination of these factors in addition to the age of onset for these events. In this regard, changes in mtDNA copy number and/or deletions seem to be age-dependent, for the clinical onset of mtDNA depletion is typically in infancy or early childhood whereas multiple deletions seldom present before adolescence. A genetic background, in combination with other genetic (originated from parental genomic DNA or maternal mtDNA) and/or epigenetic factors, for example, dysfunctional electron transport chain [15], low levels of antioxidant enzymes bound to mtDNA [67], environmental factors [49] and dietary deficiencies [68], acting additively or synergistically may lead to more damaged mtDNA during vulnerable windows such as the perinatal periods. This altered process may be causative per se, or may set up the stage for a heightened susceptibility for further insults, which may ultimately alter an appropriate development of energy status and increase autism risk. Structural instability of mtDNA, consisting either of large-scale rearrangements, tissue-specific depletion or deletions, is a major cause of mitochondrial dysfunction and disease in humans [4], and possibly in children with autism.

## Abbreviations

ADI-R: Autism Diagnostic Inventory-Revised; ADOS: Autism Diagnostic Observation Schedule; AP: Apurinic/apyrimidinic sites; 8oxodG: 8-oxo-7,8-dihydro-2′-deoxyguanosine; ASD: Autism spectrum disorders; AU: Full autism; DSM-IV-TR: Diagnostic and Statistical Manual of Mental Disorders-Fourth Edition (Text Revision); H: Heavy; ICD-10: International Statistical Classification of Diseases and Related Health Problems 10th Revision; L: Light; mtDNA: Mitochondrial DNA; mtSSB: Mitochondrial single stranded-DNA binding proteins; PBMC: Peripheral blood monocytic cells; PCR: Polymerase chain reaction; Polγ: Mitochondrial DNA polymerase gamma; ROS: Reactive oxygen species; TD: Typically developing.

## Competing interests

The authors of this publication declare that they have no conflicting financial interest in relation to the work described.

## Authors’ contributions

EN has been involved in statistical analyses of the data and drafted the manuscript; SW carried out all experiments and helped to draft the manuscript; CG conceived the study, contributed to the analysis and interpretation of data, and revised it critically for important intellectual content. All authors have given final approval of the version to be published.

## Supplementary Material

Additional file 1 **Additional material for this study has been provided under Additional documentation.** This file includes all demographics of subjects utilized in this study, sequence variants data and associated references. Click here for file
