# Supplementary material for: Evidence of reactive oxygen species-mediated damage to mitochondrial DNA in children with typical autism
Source: Mol Autism. 2013 Jan 25;4:2. doi: 10.1186/2040-2392-4-2 (PMC3570390; doi:10.1186/2040-2392-4-2)
Supplement: Additional file 1 — Additional material for this study has been provided under Additional documentation. This file includes all demographics of subjects utilized in this study, sequence variants data and associated references. [file 2040-2392-4-2-S1.pdf]

Supplementary Material

Supplementary Table1
Demographic characteristics of CHARGE participants with autism and typical development

	Samples for mtDNA deletions				Samples for mtDNA sequencing			
	**AU (*n*=67)**		**TD* (*n*=46)**		**AU (*n*=10)**		**TD* (*n*=10)**	
**Characteristic**	**Mean**	**SD**	**Mean**	**SD**	**Mean**	**SD**	**Mean**	**SD**
Maternal age, y†	36.5	4.4	35.5	5.1	37.1	3.3	37.5	5.0
Paternal age, y†	38.5	5.3	38.1	5.7	40.1	3.1	41.1	6.9
Child's age, m†	45.1	9.4	42.5	8.0	45.0	7.7	46.0	5.8
	**%**		**%**		**%**		**%**	
Maternal education								
No Bachelor degree	51.0		41.0		40.0		10.0	
Bachelor degree or higher	49.0		59.0		60.0		90.0	
Child's sex								
Male	86.5		87.2		90.0		90.0	
Female	13.5		12.8		10.0		10.0	
Child's birth year								
1998-2001	30.8		38.5		40.0			
2002-2005	69.2		61.5		60.0		100.0	
2006-2007								
Child's ethnicity								
White, non-Hispanic	46.2		46.2		90.0		40.0	
Non-White, non-Hispanic	11.5		20.5				40.0	
Hispanic	42.3		33.3		10.0		20.0	

*includes 1 control who scored 112 total on Mullen's Scales of Early Development for whom the Vineland Adaptive Behavior Scales were not completed.
†Age at entry into CHARGE Study

Supplementary Table 2
Clinical characteristics of CHARGE participants with autism and typical development

	Samples for mtDNA deletions				Samples for mtDNA sequencing			
	**AU (*n*=67)**		**TD* (*n*=46)**		**AU (*n*=10)**		**TD* (*n*=10)**	
**Assessment**	**Mean**	**SD**	**Mean**	**SD**	**Mean**	**SD**	**Mean**	**SD**
ADI-R								
Social Interaction	18.6	4.6			17.3	4.7		
Communication								
Verbal	15.5	2.7			14.6	2.4		
Non-Verbal	10.7	2.1			10.7	1.2		
Stereotyped Behaviors	6.5	2.2			7.0	1.4		

ADOS Module 1								
Communication	5.7	1.3			4.7	1.2		
Social Interaction	9.8	1.8			9.3	1.5		
ADOS Module 2								
Communication	6.5	1.5			6.0	1.2		
Social Interaction	9.5	1.5			9.4	0.5		
ADOS Module 3								
Communication	2.0							
Social Interaction	9.0							
Mullen								
Visual Reception	29.7	13.2	56.2	11.0	37.0	17.1	60.0	8.4
Fine Motor	28.0	11.9	53.0	12.4	34.9	18.1	52.6	16.2
Receptive Language	26.9	11.5	50.6	12.9	31.0	13.0	55.3	14.3
Expressive Language	26.5	10.8	52.5	11.8	31.1	9.8	52.1	12.5
Composite	61.3	18.1	106.2	18.1	70.7	20.7	110.0	20.4
SCQ			2.8	2.5			2.6	3.1
Vineland								
Communication	68.4	17.4	104.5	14.1	81.9	22.2	106.7	13.6
Daily Living Skills	64.5	11.0	101.6	13.6	74.8	14.3	94.1	14.3
Socialization	67.8	12.6	105.6	14.4	76.2	14.0	98.8	9.6
Motor Skills	72.8	17.2	105.9	14.9	84.6	17.2	104.8	17.1
Composite	63.6	13.8	105.8	14.8	75.5	18.0	101.4	16.4

*includes 1 control who scored 112 total on Mullen's Scales of Early Development for whom the Vineland Adaptive Behavior Scales were not completed.

Supplementary Table 3: *mtDNA sequence variants in PBMC from children with typical autism*

Case number	Base position	Protein	Nucleotide change	Amino acid change	mtDNA Base Substitution Diseases	Polymorphism*
1	8269	COX2	G → A	nc (Ter-Ter)		1.37%
	14927	CYTB	A → G	T-A		0.55%
2	**3197**	RNR2	T → C	nc (rRNA 16S)	(G3196A ADPD)	3.60%
	**9477**	COX3	G → A	V-I**	Thyroid tumor	3.60%
	9548	COX3	G → A	syn		0.96%
3	3480	ND1	A → G	syn	Prostate tumor	4.84%
	3552	ND1	T → C	syn		0.15%
	9055	ATP6	G → A	A-T**	PD protective factor [1]	4.96%
	9698	COX3	T → C	syn		5.10%
4	8697	ATP6	G → A	syn	Thyroid tumor	4.7%
	14905	CYTB	G → A	syn		5.1%
5	**4491**	ND2	G → A	V-I		0.67%
6	**3197**	RNR2	T → C	nc (rRNA	(G3196A ADPD)	3.60%

				16S)		
	9031	ATP6	C → T	L(2)-L(1)		NR
	**9477**	COX3	G → A	V-I**	Thyroid tumor	3.60%
7	8765	ATP6	C → T	A-V		NR
8	**4491**	ND2	G → A	V-I		0.67%
	<u>4580</u>	ND2	G → A	syn	Pancreatic cancer cell line	2.84%
	5195	ND2	C → T	syn		NR
9	3394	ND1	T → C	Y-H	LHON, NIDDM, CPT deficiency; acute leukemia platelets, leukocytes & bone marrow	1.46%

All sequence variants were homoplasmic. *From [2] and MITOMAP database[3]; NR never reported in any of these databases. ** Natural amino acid variants (from Expasy database). Numbers in bold are present more than once in cases. Underlined numbers represent somatic mutations also present in TD. Abbreviations: nc, noncoding; ADPD, Alzheimer's disease and Parkinson's disease; CPT, carnitine palmitoyl-transferase II (CPT II) deficiency; LHON, Leber hereditary optic neuropathy; NIDDM, Non-insulin dependent diabetes mellitus; PD, Parkinson's disease. Case 10 resulted identical to the reference sequence, and it was excluded from the Table.

Supplementary Table 4: *mtDNA sequence variants in PBMC from typical neurodeveloping children*

Case number	Base position	Protein	Nucleotide change	Amino acid change	mtDNA Base Substitution Diseases	Polymorphism*
1	<u>4580</u>	ND2	G → A	syn	Pancreatic cancer cell line	2.84%
	4917*	ND2	A → G	N-D	LHON/Insulin Resistance/ AMD/NRTI-PN	4.78%
	5147	ND2	G → A	syn		4.99%
	<u>8697</u>	ATP6	G → A	syn	Thyroid tumor	4.7%
2	8744	ATP6	T → C	V-A		NR
3†	3547	ND1	A → G	I-V		0.81%
	4977	ND2	T → C	syn		0.74%
	9097	ATP6	A → G	I-V**		0.04%, NR
4	3398	ND1	T → C	M-T	DMDF+HCM/GDM/ possibly LVNC-associated	0.18%
	3447	ND1	A → G	syn		0.44%
	3990	ND1	C → T	syn		0.41%

	**4529**	ND2	A → T	syn		1.22%
	8107	COX2	A → G	syn		NR
	**8251**	COX2	G → A	syn		5.3%
	**15043**	CYTB	G → A	syn	MDD-associated	28.7%
5	3316	ND1	G → A	A-T	NIDDM/LHON/PEO	0.78%
	3453	ND1	C → T	syn		R
	3465	ND1	A → T	M-I		R
	4167	ND1	C → T	syn		0.07%
	8701	ATP6	A → G	T-A**	Thyroid tumor	34.5%
	9540	COX3	T → C	syn		34.9%
	9545	COX3	A → G	syn		2.0%
	**15043**	CYTB	G → A	syn	MDD-associated	28.7%
	15109	CYTB	T → C	syn		0.04%, NR
	15301	CYTB	G → A	syn		32.0%
6	**4529**	ND2	A → T	syn		1.22%
	8155	COX2	G → A	syn		0.18%
	**8251**	COX2	G → A	syn		5.3%
	15003	CYTB	G → C	G-A		NR
	**15043**	CYTB	G → A	syn	MDD-associated	28.7%
7	15212	CYTB	A → G	I-V		0.04%
	15218	CYTB	A → G	T-A		1.4%
8	14905	CYTB	G → A	syn		5.1%
9	4646	ND2	T → C	syn	Glioblastoma	0.56%

All mutations were homoplasmic. *From [2]. [†] This sample contained in addition a 9 bp deletion from nt 8281 to 8289 (A[delCCCCCTCTA]G) consistent with haplogroup B according to [3]. For this same case, the nucleotide change was reported as a polymorphism in one database but not reported in MITOMAP. Numbers in bold are present more than once in TD. Underlined numbers represent somatic mutations also present in AU. ** Natural amino acid variants (from Expasy database). NR, not reported in any mitochondrial database. R, reported in MITOMAP but not in ref. [2]. Abbreviations: AMD, age-related macular degeneration; DMDF, diabetes mellitus & deafness; GDM, gestational DM; HCM, hypertrophic cardiomyopathy; LVNC, left ventricular noncompaction; MDD, major depressive disorder; NRTI-PN, antiretroviral therapy-associated peripheral neuropathy. Case 10 sequence was identical to the reference one. *haplogroup T marker.

REFERENCES

1. van der Walt JM, Nicodemus KK, Martin ER, Scott WK, Nance MA, Watts RL, Hubble JP, Haines JL, Koller WC, Lyons K, et al: **Mitochondrial polymorphisms significantly reduce the risk of Parkinson disease.** *American journal of human genetics* 2003, **72:**804-811.

2. Ingman M, Gyllensten U: **mtDB: Human Mitochondrial Genome Database, a resource for population genetics and medical sciences.** *Nucl Acids Res* 2006, **34:**D749-751.
3. Brandon MC, Lott MT, Nguyen KC, Spolim S, Navathe SB, Baldi P, Wallace DC: **MITOMAP: a human mitochondrial genome database--2004 update.** *Nucleic Acids Res* 2005, **33:**D611-613.
